# Retinoic acid regulates olfactory progenitor cell fate and differentiation

**DOI:** 10.1186/1749-8104-8-13

**Published:** 2013-07-05

**Authors:** Marie Paschaki, Laura Cammas, Yuko Muta, Yoko Matsuoka, Siu-Shan Mak, Monika Rataj-Baniowska, Valérie Fraulob, Pascal Dollé, Raj K Ladher

**Affiliations:** 1 Laboratory for Sensory Development, RIKEN Center for Developmental Biology, Kobe 650-0047, Japan; 2 Institut de Génétique et de Biologie Moléculaire et Cellulaire (IGBMC), UMR 7104 CNRS, U 964 INSERM, Université de Strasbourg, B.P. 10142, 67404, Illkirch Cedex, France

**Keywords:** Olfactory neurons, Neuronal differentiation, RALDH, Retinoid signaling, Sensory systems, Stem cells

## Abstract

**Background:**

In order to fulfill their chemosensory function, olfactory neurons are in direct contact with the external environment and are therefore exposed to environmental aggressive factors. Olfaction is maintained through life because, unlike for other sensory neuroepithelia, olfactory neurons have a unique capacity to regenerate after trauma. The mechanisms that control the ontogenesis and regenerative ability of these neurons are not fully understood. Here, we used various experimental approaches in two model systems (chick and mouse) to assess the contribution of retinoic acid signaling in the induction of the olfactory epithelium, the generation and maintenance of progenitor populations, and the ontogenesis and differentiation of olfactory neurons.

**Results:**

We show that retinoic acid signaling, although dispensable for initial induction of the olfactory placode, plays a key role in neurogenesis within this neuroepithelium. Retinoic acid depletion in the olfactory epithelium, both in chick and mouse models, results in a failure of progenitor cell maintenance and, consequently, differentiation of olfactory neurons is not sustained. Using an explant system, we further show that renewal of olfactory neurons is hindered if the olfactory epithelium is unable to synthesize retinoic acid.

**Conclusions:**

Our data show that retinoic acid is not a simple placodal inductive signal, but rather controls olfactory neuronal production by regulating the fate of olfactory progenitor cells. Retinaldehyde dehydrogenase 3 (RALDH3) is the key enzyme required to generate retinoic acid within the olfactory epithelium.

## Background

To fulfill their chemosensory role, olfactory neurons contact the external environment, making them prone to damage. The olfactory epithelium (OE) is able to replace damaged olfactory neurons throughout life. This epithelium derives from the olfactory placode (OP), found within the non-neural ectoderm at the rostral tip of the embryo. It comprises olfactory receptor neurons (ORNs) and non-neuronal subtypes, such as sustentacular cells and their progenitors (for a scheme of the OE, see Additional file 1: Figure S1). ORNs derive from olfactory stem cells by a multistep process involving precursors expressing the basic helix-loop-helix factor Ascl1, whose mutation results in a failure of olfactory neurogenesis. Ascl1 is required for induction of the atonal-related gene *Neurogenin1* (*Neurog1*) in immediate neuronal precursors (INPs), which then differentiate into ORNs expressing β-III-tubulin [1]. The OE of *Ascl1-*null mouse mutants contains excessive numbers of precursors expressing *Sox2* and *Ascl1*[2], indicating that *Ascl1* loss-of-function prevents their transition into more differentiated cell types.

While the identity of the olfactory stem cell upstream to the Ascl1-expressing progenitor is unclear, a cell type co-expressing Sox2 and Pax6 is a good candidate. These factors are typically associated with multipotent cell types in developing neuroepithelia, and in the OE are expressed in a subset of basal cells that do not express Ascl1 [3]. Acute OE damage by methyl bromide-induced lesion or by olfactory bulbectomy, causes Pax6 upregulation before Ascl1 upregulation [4]. The mechanisms controlling the transition between the different cell states are poorly understood, although computational modeling suggests that secreted factors exert exquisite feedback control on different cell populations to ensure a rapid and regulatable response to injury [3]. An interplay between the Tgfβ ligand GDF11, the activin-binding protein follistatin, and activin/inhibinβB, has been described as regulating the balance between stem/progenitor cells and INPs, and possibly the choice between neuronal and sustentacular cell fates [5].

Retinoic acid (RA), a metabolite of vitamin A (retinol), is a signaling molecule involved in various developmental processes (refs. [6-8] for reviews). It is synthesized by cell populations expressing one of three retinaldehyde dehydrogenases (RALDH1, 2, 3, whose corresponding genes are also known as Aldh1a1-a3) and, by acting as a ligand for nuclear receptors, stimulates the transcriptional activity of target genes ([9] for a review). Experiments involving murine OE-mesenchymal co-cultures implicated RA as one of the signals involved in OE patterning and/or differentiation [10,11]. However, it is unclear if RA is merely an inductive signal or a neuronal differentiation factor, and/or to what extent RA signaling affects olfactory neurogenesis. Here, we show that retinoid signaling is not required for induction of the OP, but it is a key factor regulating the progression of olfactory neural progenitors into more committed precursors. RA depletion using pharmacological inhibition, or mutation of its synthesizing enzyme RALDH3, results in a failure of progenitor maintenance, and this depletion leads to a diminished capacity of olfactory precursor cells to renew and differentiate into ORNs.

## Results and discussion

### Retinoic acid is not necessary for olfactory placode induction

**Figure 1:**
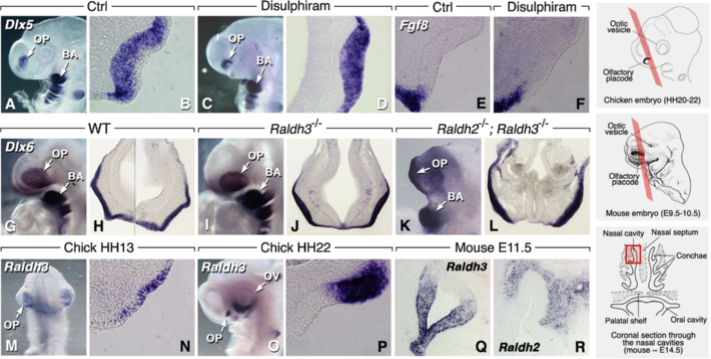
**Retinoic acid and olfactory induction. (A**-**F)** Chicken embryos treated with disulphiram-impregnated beads (100 μM in DMSO) during OP induction (HH-stage 14) show unaffected placodal development (**A**,**C**: whole-mounts; **B**,**D**: frontal sections after *Dlx6* labeling) and *Fgf8* expression in the placodal rim **(E**,**F)**. **(G**-**L)***Raldh3*^−/−^ mouse mutants **(I**,**J)** and *Raldh2*^−/−^; *Raldh3*^−/−^ compound mutants **(K**,**L)** exhibit a *Dlx5*-labeled olfactory placode at E9.5 (**G**,**I**,**K**: whole-mounts; **H**,**J**,**L**: transverse sections). **(M**-**R)***Raldh3* is expressed within chicken **(M**-**P)** and mouse **(Q)** early embryonic OE. *Raldh2* appears later (E11.5 in mouse) in surrounding mesenchyme **(R)**. On the right are schemes explaining the section planes used throughout this study. Chicken and mouse embryos were sectioned according to a frontal section plane, as suggested by the colored Z-plane shown in perspective. For older mouse embryos and fetuses (**Q**,**R**, and subsequent figures), a detail of one of the nasal cavities is shown, as suggested by the boxed area in the bottom scheme. Mouse schemes are adapted from [23].

Grafting and co-culture experiments in amphibian embryos suggested that anterior mesendoderm and forebrain are the sources of inducing signals for the OPs (refs. [12-14] for reviews). As *Raldh* genes are expressed in forebrain neuroepithelium and facial mesenchyme/ectoderm during OP induction [15-17], RA is a candidate molecule for this process. We used the chick and mouse models to investigate whether endogenous RA depletion affects OP induction. In chick, the OP is identified by expression of the distal-less family gene *Dlx6* (Figure 1A, B). Embryos were treated prior to OP induction with beads soaked with disulphiram, an inhibitor of RALDHs, as previously described [18]. These treatments did not alter *Dlx6* expression pattern in the developing OP (Figure 1A-D). FGF8 is thought to initiate a morphogenetic center at the rim of the invaginating OP [19], and reciprocal inhibitory interactions between RA and FGF8 have been described in other embryonic regions [20-22]. We investigated whether inhibiting RA synthesis affects *Fgf8* expression at the ectoderm/placodal rim. *Fgf8* transcripts were normally distributed in disulphiram-treated embryos, indicating that the presence of RA is not required to restrict *Fgf8* expression at the placodal rim (Figure 1E, F).

To assess the requirement of RA in placodal induction, we used mouse mutants for RA-synthesizing enzymes. Although *Raldh3* is prominently expressed in pre-placodal ectoderm [16,17], *Raldh3*^−/−^ null mutants displayed a well-developed, *Dlx5*-labeled OP at embryonic day (E)9.5 (Figure 1G-J). Despite their facial and forebrain hypoplasia, *Raldh2*^−/−^; *Raldh3*^−/−^ double-null mutants also exhibited a *Dlx5*-labeled placode comparable in size to control or *Raldh3*^−/−^ mutants (Figure 1K, L). Collectively, these results indicate that a deficiency in RA signaling, resulting from either gene disruptions or pharmacological inhibition of its synthesizing enzymes, does not prevent early inductive steps of the OP.

### Retinoic acid activity in olfactory placode epithelium is RALDH3-dependent

Expression of *Raldh* genes during olfactory development has mainly been studied in mouse (e.g., [24-26]), although Blentic et al. also reported expression of *Raldh3* in the chick embryonic olfactory placode [27]. In agreement with these authors we found that, among *Raldh* genes, only *Raldh3* is expressed in the chick OP epithelium (Figure 1M, N), with expression intensifying and soon becoming restricted towards its rostrolateral part (Figure 1O, P). Regionalized expression has been previously described for *Raldh3* in the mouse OP [16]. Here, *Raldh3* expression becomes restricted towards the rostrolateral epithelium, throughout mid-late gestational stages (Figure 1Q). Neither *Raldh1* nor *Raldh2* transcripts were detected in the OE (data not shown; [24]), although *Raldh2* was detected in surrounding mesenchyme from E11.5 (Figure 1R).

**Figure 2:**
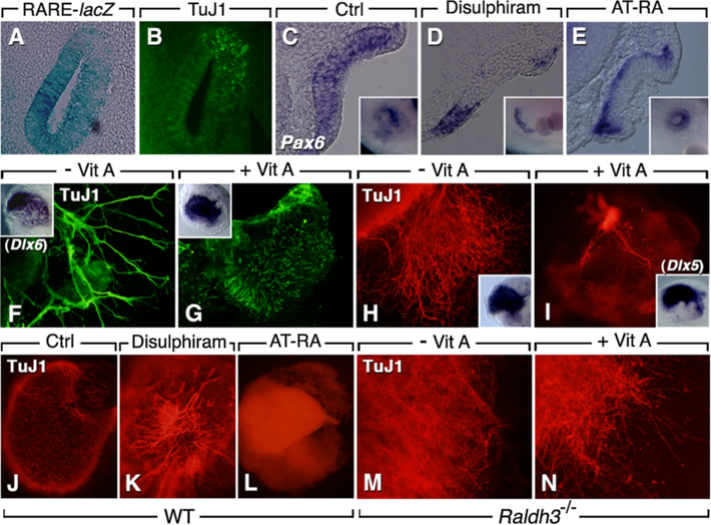
**Retinoic acid signaling affects olfactory neural differentiation. (A**,**B)** Endogenous RA activity, detected by the RARE-*lacZ* reporter transgene in E11.5 mouse OE, spatially segregated with differentiating (TuJ1-labeled) neurons. **(C**-**E)** Disulphiram bead administration to HH14 chick embryos leads to an absence of Pax6+ progenitors in most of the OP **(D)**, whereas all-*trans*-RA (AT-RA) treatment leads to a basal location of Pax6+ cells **(E)**. **(C)** Control embryo (DMSO soaked bead). Main panels: transverse sections; insets: whole-mount views. **(F**,**G)** Culture of HH14 chick OPs in medium devoid of vitamin A stimulates neurite outgrowth from differentiating (TuJ1+) ORNs. **(H**,**I)** A similar effect is seen in E10.5 mouse olfactory explants. Insets: explants labeled with *Dlx6***(F**,**G)** or *Dlx5***(H**,**I)** after culture, showing that the OE developed similarly in both conditions. **(J**-**L)** Culture of E10.5 mouse OE in presence of disulphiram strongly increases ORN neurite outgrowth **(K)**, whereas AT-RA treatment has an inhibitory effect **(L)**, in comparison to the control (DMSO-treated) explants **(J)**. **(M**,**N)** Explants from E10.5 *Raldh3*^−/−^ embryos form differentiating and neurite-extending ORNs (TuJ1-labeled), whether cultured in absence **(M)** or presence **(N)** of vitamin A.

Transcriptional activity of RA was demonstrated within the differentiating OE by analysis of retinoic acid response element (RARE)-*lacZ* reporter transgenic mice (Figure 2A). Interestingly, analysis of serial sections revealed that β-III-tubulin-positive neurons first appear in the lateral placodal region, essentially devoid of RARE-*lacZ* reporter activity (Figure 2B), suggesting that the presence of RA may prevent neuronal differentiation. There was no RARE-*lacZ* reporter activity in the OE of E10.5-E12.5 *Raldh3*^−/−^ mice (Additional file 2: Figure S2; [28]), implicating RALDH3 as the critical RA-synthesizing enzyme within this neuroepithelium.

### Retinoic acid affects olfactory neurogenesis in both chick and mouse

It has been previously reported that RA treatments of olfactory explants reduce neuronal differentiation [10,29]. However, RA treatment of immortalized cell lines derived from mouse OP has been shown to induce the maturation of ORNs [30]. To investigate the role of endogenous RA, we administrated disulphiram by bead implantation under the OP of Hamburger-Hamilton stage (HH)14 chicken embryos, and used *Pax6* as a marker of olfactory stem cells (Figure 2C, D). Disulphiram administration led to an absence of *Pax6*-expressing cells in a large area of the placode, with *Pax6*+ cells found only along the placodal rim (Figure 2D). Interestingly, all-*trans*-RA (AT-RA) bead administration resulted in a basal location of *Pax6*-expressing cells (Figure 2E). These results suggest that RA signaling promotes the production and/or the maintenance of the *Pax6*+ progenitor cell population in the placodal epithelium.

To address the role of RA in neuronal differentiation, we adopted an explant approach; HH14 chick OPs were cultured for 48 h in serum-free media in presence or absence of vitamin A (retinyl acetate), and neuronal development was assessed using β-III-tubulin (TuJ1) staining. In the absence of vitamin A, robust differentiation was observed in explants, with long axons typical of olfactory neurons (Figure 2F). When vitamin A was included, TuJ1-positive cells did not have olfactory neuronal morphology, showing only small processes that failed to emigrate from the explant (Figure 2G). Similarly, culturing wild-type E10.5 mouse OE in the absence of vitamin A stimulated neuronal differentiation, while culturing these explants with vitamin A strongly suppressed ORN differentiation (Figure 2H, I). Culturing E10.5 OE for two days in the presence of disulphiram led to a marked increase in neurogenesis (Figure 2K), in comparison to control conditions (Figure 2J). Conversely, a 2-day treatment with AT-RA inhibited ORN formation (Figure 2L). Explants from E10.5 *Raldh3*^−/−^ embryos were insensitive to vitamin A supplementation, showing neuronal differentiation whether cultured in the presence (Figure 2N) or absence (Figure 2M) of vitamin A. Collectively, these data show that exogenous vitamin A and AT-RA have inhibitory effects on ORN differentiation in the wild-type embryonic OE, and that RALDH3 deficiency affects the potential of this epithelium to produce differentiated neurons.

### RA regulates proliferating progenitors and olfactory neuron precursors

**Figure 3:**
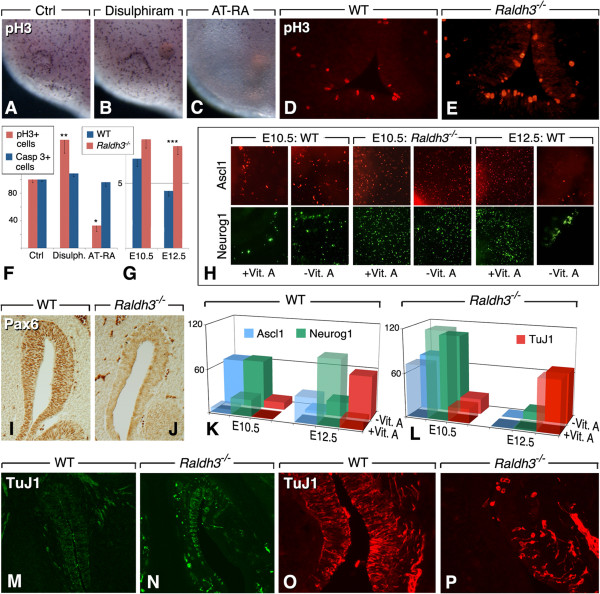
**Retinoid deficiency affects the olfactory neuronal lineage. (A**-**C)** Treatment of HH14 stage chicken embryos with disulphiram **(B)** or AT-RA **(C)** beads impacts positively or negatively on OP mitotic (pH3-labeled) cells. **(D**,**E)** pH3 immunodetection in the OE of E12.5 wild-type and *Raldh3*^−/−^ mouse embryos, showing an increased number of labeled cells in the mutant. **(F)** Quantification of pH3+ (mitotic) or activated-caspase 3+ (apoptotic) cells in disulphiram or AT-RA-treated HH14 chicken OPs (n = 6 embryos/condition, expressed as percentages with respect to controls; *: *p* <0.05; **: *p* <0.01). **(G)** Quantification of pH3+ cells in the OE of E10.5 or E12.5 wild-type and *Raldh3*^−/−^ mouse embryos (n = 6 embryos/genotype; three sections of the OE were counted for each embryo; ***: *p* <0.001). **(H)** Examples of immunofluorescence detection of Ascl1 (top panels) or Neurog1 (bottom panels), after culturing E10.5 or E12.5 murine olfactory explants for 48 h in defined medium, in the presence (+Vit. A) or absence (−Vit. A) of vitamin A. The corresponding data are quantified in **K**,**L**. **(I**,**J)** Pax6 immunodetection in the OE of E12.5 wild-type and *Raldh3*^−/−^ embryos. **(K**,**L)** Quantitative analysis of neuronal progenitor populations in murine olfactory explants collected at E10.5 (left-side histograms) or E12.5 (right-side histograms), from wild-type **(K)** or *Raldh3*^−/−^**(L)** mice, and cultured for 48 h in medium containing (+Vit. A, front row) or devoid of (−Vit. A, back row) vitamin A (n = 3 explants per stage and genotype; Additional file 3: Table S1 for statistics). Explants were analyzed by immunofluorescence using anti-Ascl1, -Neurog1, and/or TuJ1 antibodies. **(M**-**P)** TuJ1 immunodetection in the OE of E12.5 wild-type **(M,O)** and *Raldh3*^−/−^**(N**,**P)** mice. **(O**,**P)** are higher magnifications of the distal OE, taken from specimens labeled with a different fluorochrome.

Since modulation of the RA pathway appears to regulate the pool of *Pax6*+ progenitor cells, and considering that olfactory neurogenesis is inhibited by RA *in vitro*, we wondered at which step of the ORN lineage RA may act. We first assessed if modulation of RA signaling affects apoptotic cell death or progenitor cell proliferation in the OE. Apoptotic cells, identified by activated caspase-3 immunolabeling, were not significantly changed in the OE of chicken embryos, 48 h after disulphiram or AT-RA bead implantation (Figure 3F). However, disulphiram administration increased phospho-histone H3 (pH3)-labeled proliferative cells (Figure 3A, B, F), and AT-RA inhibited cell proliferation (Figure 3C, F). An effect of RA deficiency on cell proliferation was also observed in *Raldh3*^−/−^ mouse mutants, with a slight increase of pH3-labeled mitotic progenitors in the OP at E10.5 (Figure 3G), and a more pronounced increase in the OE of E12.5 mutant embryos (Figure 3D, E, G).

These observations prompted us to analyze progenitor cell populations in the OE of *Raldh3*^−/−^ mutants. Olfactory neurogenesis occurs through distinct steps, marked by the expression of particular transcription factors. Ascl1 marks a fast-proliferating neuronal progenitor cell, which will generate an immediate neural precursor (INP) expressing Neurog1 [5,31]. Upstream of the Ascl1-positive neuronal progenitor lies a slow-proliferating, Pax6-expressing progenitor cell, postulated to be the resident stem cell population of the OE [32,33]. At E12.5, numerous Pax6-expressing cells are found throughout the OE of wild-type mice (Figure 3I), whereas Pax6-positive cells were diminished in the OE of *Raldh3*^−/−^ mutants (Figure 3J), supporting the hypothesis that RA is required to maintain the Pax6-positive progenitor cell population within this epithelium.

To investigate how olfactory neuronal progenitors are affected by retinoids, we used the explant culture system. Wild-type OE were collected at two stages (E10.5, E12.5; n = 3 for each stage), and cultured for 48 h in minimal medium containing or devoid of vitamin A. Ascl1-positive and Neurog1-positive cells (Figure 3H) were scored after culture, as were olfactory neurons identified by TuJ1 staining (Figure 3K, L). For statistical analysis see Additional file 3: Table S1. The chosen stages correspond to two phases of neurogenesis; at E10.5, primary neurogenesis generates relatively few neurons thought to be pioneers establishing the terminal nerve ganglion [19]. From E12.5, a more sustained wave of neuronal differentiation (“established” neurogenesis) predominates. Culturing E10.5 OE in vitamin A-depleted medium resulted in the presence of higher Ascl1+ and Neurog1+ progenitor populations (Figure 3H, left-side panels), as well as more numerous TuJ1+ neurons (Figure 3K, back line histograms), when compared to explants cultured in vitamin A-containing medium (Figure 3K: front line histograms). On the other hand, cultures of E12.5 OE showed the presence of high Ascl1+ and Neurog1+ populations in the vitamin A-supplemented condition, whereas both cell populations were markedly lower in vitamin A-devoid cultures (Figure 3H, right-side panels), which showed an increased neuronal (TuJ1+) population (Figure 3K, right-side histograms).

These data indicate that the vitamin A/retinoid status affects neuronal progenitor populations in the OE. During primary neurogenesis, an absence of retinoids (as observed for E10.5 cultures in vitamin A-depleted medium) favors the production of committed (Ascl1+, Neurog1+) precursors and increases differentiating (TuJ1+) neurons. During the later phase of established neurogenesis (E12.5 cultures), when a greater number of committed neuronal precursor cells are produced, removal of the retinoid source causes precursors to differentiate, depleting the pool of Ascl1+ precursors and highly increasing TuJ1+ differentiating ORNs.

Analysis of *Raldh3*^−/−^ olfactory explants cultured under the same conditions revealed that during primary neurogenesis (E10.5) the populations of Ascl1+ and Neurog1+ precursors were high in both vitamin A-supplemented and devoid conditions (Figure 3H, middle panels, and Figure 3L, left side histograms). Strikingly, in E12.5 cultures, the numbers of Ascl1+ and Ngn1+ progenitors collapsed, irrespective of the presence of vitamin A, whereas both culture conditions led to the presence of high numbers of TuJ1+ neurons (Figure 3L, right-side histograms). These high numbers, together with the depletion of Ascl1+ and Neurog1+ populations, could reflect massive differentiation occurring at the expense of progenitor self-renewal or maintenance. This supports the idea that RA is necessary to maintain progenitor populations within the developing OE.

To assess how changes in these populations may affect olfactory neurogenesis *in vivo*, we analyzed TuJ1-positive neurons on sections of E12.5 *Raldh3*^−/−^ embryos. The OE in mutants exhibited increased TuJ1 staining, suggesting the presence of higher numbers of differentiating neurons (Figure 3M, N). Mutants also showed disorganization of the distal OE, with few disperse differentiating neurons at this level (Figure 3O, P).

### RA is necessary to sustain olfactory neurogenesis

**Figure 4:**
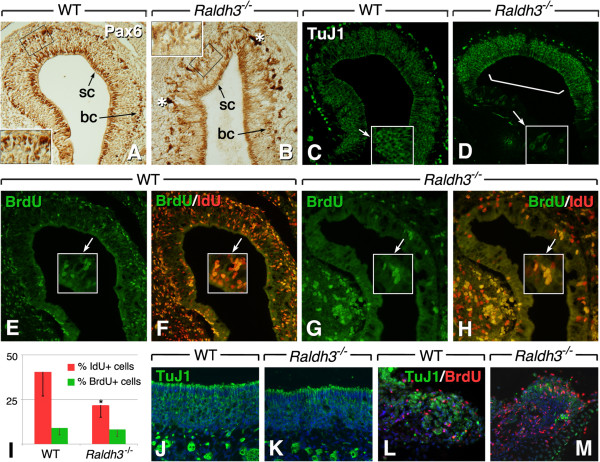
**Retinoic acid is required for sustained olfactory neurogenesis. (A**,**B)** Pax6 immunochemical detection on sections of the OE of E17.5 wild-type **(A)** and *Raldh3*^−/−^**(B)** embryos. Insets show details of the basal region of the epithelium (boxed) at a higher magnification. Asterisks point to dark stained areas probably due to artifactual trapping of the immunochemical reaction product. **(C**,**D)** TuJ1 immunofluorescence on sections of the OE of E17.5 wild-type **(C)** and *Raldh3*^−/−^**(D)** embryos. While numerous TuJ1+ neurons are seen along the recess of the ethmoturbinate cavity in the mutant (bracket in **D**), neurons are markedly depleted in more distal regions of the epithelium (compare insets in **C** and **D**). **(E**-**I)** Detection of fast- and slow-proliferating cells in E17.5 OE after IdU/BrdU injections at E15.5/E16.5 (wild-type: **E**,**F**; *Raldh3*^−/−^: **G**,**H**). **(E**,**G)** BrdU labeling (green). **(F**,**H)** Merged labeling, IdU+/BrdU- cells appearing orange to yellow. Insets show higher magnifications of selected regions. **(I)** Quantification of IdU+ and BrdU+ cells (n = 3 animals/genotype; *: *p* <0.05). **(J**-**M)** Analysis of neuronal regeneration in E17.5 olfactory explants (wild-type: **J**,**L**; *Raldh3*^−/−^: **K**,**M**) cultured for 48 h in neurobasal medium containing vitamin A, with BrdU added during the first 24 h. **(J,K)** Explants at the start of culture. **(L**,**M)** Explants at 48 h. TuJ1 (green), BrdU (red), DAPI (blue). sc: sustentacular cells; bc: basal cells.

Considering the effects of RA depletion on olfactory neurogenesis at early embryonic stages, we then investigated its consequences at a late neurogenic stage (E17.5). We first analyzed the distribution of Pax6+ progenitor cells and TuJ1+ ORNs in *Raldh3*^−/−^ mutants. An abnormal distribution of Pax6-expressing cells was observed, with Pax6+ cells mainly marking sustentacular cells at the apical side, whereas fewer Pax6+ basal progenitor cells were observed in comparison to wild-type (Figure 4A, B). This again suggests that Pax6+ progenitor cells are not properly maintained in the absence of RA. In mutants, TuJ1 immunostaining indicated a preferential location of olfactory neurons in the deeper regions of the ethmoturbinates (bracket in Figure 4D), whereas more distal regions were mainly TuJ1-negative (compare Figure 4C and D). These data support the hypothesis of a diminished olfactory progenitor pool in the *Raldh3*^−/−^ OE, eventually exhausting its capacity to generate new differentiating ORNs distally at late gestational stages.

To analyze the proliferative capacity of stem/progenitor cells, we injected iododeoxyuridine (IdU) maternally at E15.5, followed by BrdU injection 20 h later. Embryos were collected at E17.5 in order to distinguish fast and slow proliferative cells (the former having integrated both IdU and BrdU, the latter integrating only IdU). We found that, in mutants, overall proliferation was decreased by over 50% within the OE (Figure 4E-H). Interestingly, labeled cells in mutants were located basally (Figure 4H), in contrast with the more widespread pattern of proliferating cells in wild-type (Figure 4F), indicating that at late developmental stages RA is necessary to maintain the proliferative potential of the progenitor cell population. More importantly, when quantified separately, only the IdU+ slow proliferating cells were significantly diminished, while the numbers of BrdU+ cells were comparable (Figure 4I).

Basally located slow-proliferating cells are postulated to be the stem cell population that is activated during extreme damage, such as chemical trauma or axonectomy, to regenerate the OE [32,33]. As *Raldh3*^−/−^ mice show an early post-natal lethality [28], whole animals could not be used to assess the effect of a lack of RA in the post-natal OE. Instead, we turned to late-stage explant cultures [5]. Explantation of the OE causes the olfactory nerve to be sectioned, leading to neuronal death. Such a paradigm allowed us to investigate the renewal of olfactory neurons after damage. Explants were cultured in defined medium, with BrdU added at the onset of culture, and analyzed by double BrdU/TuJ1 labeling. Consistent with our *in vivo* observations, at the onset of culture, wild-type epithelium was thick and contained numerous neurons, whereas the *Raldh3*^−/−^ OE was thinner and with fewer ORNs (Figure 4J, K). After 24 h of culture, very few neurons could be detected in OE explants taken from either wild-type or mutants. However, by 48 h, significant numbers of TuJ1+ neuronal cells were observed in wild-type cultures, some of which were also BrdU+ (Figure 4L), indicating that the OE was able to produce new neurons as a response to damage. In contrast, *Raldh3*^−/−^ explants showed very few TuJ1+ neurons (Figure 4M). Furthermore, even though numerous BrdU+ cells were present, these did not co-label with neurons. Thus, although progenitor cells retain their proliferative ability in the *Raldh3*^−/−^ OE, the renewal of neurons is severely hindered.

## Conclusions

**Figure 5:**
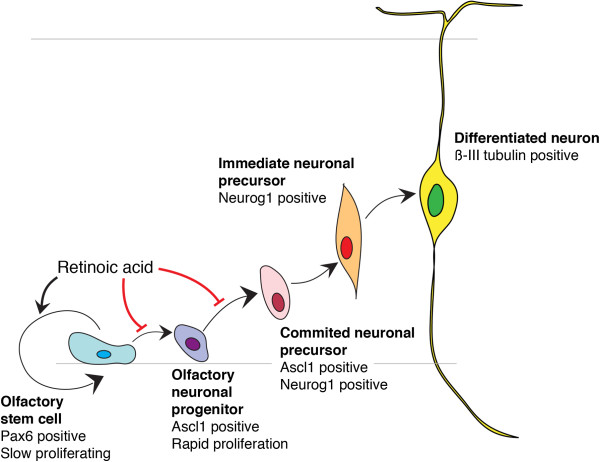
Scheme depicting the postulated role of retinoic acid signaling during olfactory neurogenesis.

The complex molecular and cellular interactions between the olfactory neuroepithelium and the underlying tissues (telencephalon and neural crest derived mesenchyme) make it difficult to dissect the specific processes and molecular interactions regulating cell fate. ORNs have the unique capacity to regenerate throughout life, while in other parts of the nervous system the neuronal regenerative capacity declines over time [34]. Our data highlight the role of RA in the process of neuronal generation and renewal within the OE. We showed that RALDH3 is the key enzyme generating RA to maintain the olfactory progenitor pool and control their differentiation potential. Our experiments indicate that RA is not involved in early induction of the OE, but is necessary for the regulation of intermediate cell populations that lead to fully differentiated ORNs. One target population is likely to be the pool of Pax6+ stem cell-like progenitors. This population is diminished in the OE of RALDH3 deficient mice and, in the absence of Pax6+ progenitor cells, Ascl+ and Neurog1+ neuronal precursors cannot be renewed. We thus propose a model in which RA signaling is required to stably maintain the slowly proliferating, Pax6+ stem/progenitor population and regulate their progression towards more committed olfactory neuronal precursors (Figure 5). In conditions of RA deficiency, the stem/progenitor cells rapidly engage into the neurogenic pathway, precociously giving rise to differentiating neurons. The transient increase in cell proliferation, observed during early stages of olfactory neurogenesis (E10.5-12.5) in *Raldh3*^−/−^ mutants, could be interpreted as a cellular attempt to fill in the diminished progenitor population. However, the pool of stem cells cannot be maintained over time, eventually leading to a depletion of the entire neuronal lineage at the late developmental stages, and an inability of the OE to (re)generate new neurons.

Another molecular pathway regulating the differentiation of olfactory progenitor cells is the Notch pathway. Recent data from the Gunhaga laboratory [35] demonstrated that an inhibition of Notch signaling in chick results in a decreased proliferation and an increased differentiation within the developing OE. We analyzed expression of Notch pathway genes (*Notch1* and *2*, *Dll1*, *Hes5*) in the developing OE, and observed no difference in the developing OE of wild-type and *Raldh3*^−/−^ embryos at E14.5 and E17.5. These observations suggest that the population of Hes5+ progenitor cells, which is Notch-dependent and is distinct from the Ascl1+ population [36], might not be altered by RA deficiency. In chick, RA has been shown to repress the formation of a neuroendocrine population (the gonadotrophin-releasing hormone or GnRH-1 neurons) arising in the early OP [37]. It will be interesting to see if this neuroendocrine population is affected in the *Raldh3*^−/−^ murine model.

Our data contribute to a better understanding of the molecular mechanisms that control olfactory neurogenesis, and indicate that RA is among the first signals to act in the process of maintenance and differentiation of the olfactory common progenitor. They also support the idea that retinoid signaling could be one of the components explaining the unique regenerative ability of the ORNs. RA has long been implicated in regenerative processes; for instance, it is able to re-specify the blastema of amphibian regenerating limbs [38], and it is among the signals critical for zebrafish heart regeneration [39]. It is thought that the lack of regenerative capacity of most mammalian tissues may result from an evolutionary “silencing” or loss of function, in adult stem/progenitor cell populations, of key signals operating during ontogenesis of these same populations (e.g., [40]). The observation that RALDH3 is specifically present in a small population of adult olfactory basal cells contributing to the neuronal lineage after chemical lesion [26], leads to the intriguing possibility that RA signaling might remain functional in olfactory stem cells throughout life, and may therefore participate in the evolutionarily conserved regenerative ability of the OE. Generation of murine models allowing to selectively ablate RALDH3 in adult olfactory stem cells will be critical to validate this hypothesis.

## Methods

### Chick and mouse embryos

Fertilized hens’ eggs (Shiroyama Farms, Kanagawa, Japan) were incubated in a humidified chamber at 38°C until the desired developmental stage. Embryos were staged according to the Hamburger and Hamilton (HH) stage series [41]. *Raldh3* null-mutant mice are as described in Dupe et al. [28]. *Raldh2*^+/−^;*Raldh3*^+/−^ mice were crossed and *Raldh3*^−/−^ embryos were compared to their wild-type littermates at several stages of pre-natal development. All procedures were approved by the local institutional animal care facilities for experiments conducted in RIKEN CDB, Japan and IGBMC, France.

### Chick embryo *ex ovo* culture and bead implantation

Chick embryos were cultured on agar-albumen culture dishes. AGI beads (Sigma) were soaked in AT-RA (10 μM in DMSO) or disulphiram (100 μM in DMSO) for at least 12 h. Control beads were soaked in DMSO. After bead implantation under the developing OP, embryos were incubated for two days at 37°C in a humidified incubator with 5% CO_2_ (EC culture, [42]), fixed in 4% paraformaldehyde (PFA) for 2 h, rinsed in phosphate-buffered saline (PBS), and processed for further analysis.

### Chick and mouse olfactory explant culture

The olfactory ectoderm was dissected from the rostral tip of HH14 chick embryos and freed from underlying tissue by soaking in 5 U/mL dispase and subsequent manual dissection. The OE was isolated from E10.5, E12.5, and E17.5 mouse embryos with a similar procedure. Controlateral (left and right) explants from the same embryos were cultured in the presence or absence of vitamin A. All experiments were repeated in triplicate.

The explants were transferred into collagen drops [43]. After gelling, the recombinant tissue was cultured in DMEM medium containing B27 supplement with or without vitamin A (both provided by GIBCO as 50× concentrated stock solutions), at 37°C for 24 h or 48 h.

For bead implantation, DMSO- (control condition), AT-RA-, or disulphiram-soaked beads were placed within the collagen drop, in direct contact with the tissue, at the same concentrations as for the chicken *ex ovo* experiments, and explants were analyzed 48 h thereafter.

### In situ hybridization and immunostaining

After fixation in 4% paraformaldehyde, samples were washed in PBS. Collagen drops were processed for in situ hybridization and immunohistochemistry as described previously [44]. A detailed protocol for in situ hybridization of mouse embryo cryosections is described at http://www.empress.har.mrc.ac.uk/browser/ (gene expression section). Antisense RNA probes specific for chicken *Dlx6*[45], *Pax6*[46], and *Fgf8*[47], and for mouse *Dlx5*[48], *Raldh2,* and *Raldh3*[25] were generated from linearized plasmid templates. Chicken *Raldh2* and *Raldh3* probes were synthesized from PCR-generated templates (adapted from [49]), using the following primers: *Raldh2* (forward: 5′GGCCGCGGAAACCAGCAGAACAAACACCACTC; reverse 5′ CCCGGGGCCCTTGATTGAAGAAGACCCCCTG) and *Raldh3* (forward 5′ GGCCGCGGGCTGAACAAACTCCTCTCACATCAC; reverse 5′ CCCGGGGCTTCTGGCATCAAAGGGGTCTCC). Probes were labeled with digoxigenin and detected using an alkaline phosphatase-coupled antibody (1:2,000, Roche) followed by NBT/BCIP (Roche) staining.

Immunohistochemistry on frozen sections or on whole embryos was performed as previously described [46]. The following primary antibodies were used: phospho-histone H3 (Upstate; 1:500) was used to indicate mitosis; an antibody recognizing the cleaved (and active) form of caspase 3 (BD Pharmingen; 1:300) was used to label apoptotic cells; antibodies against β-III tubulin (TuJ1, Covance; 1:1,000), Pax6 (Covance; 1:600), Neurog1 (Santa Cruz; 1:300), and Ascl1 (BD Pharmingen; 1:300) were used to identify olfactory cells at various stages of differentiation. Secondary antibodies were anti-rabbit Alexa 488 or anti-mouse Alexa 594 (Invitrogen; both at 1:1,000) to be visualized under fluorescence, or anti-mouse horseradish peroxidase conjugated (Dako; 1:1,000).

For proliferation analysis, pregnant mice were injected intraperitoneally with iododeoxyuridine (IdU; 100 mg/kg body weight) at E15.5, followed by a BrdU (100 mg/kg body weight) injection 20 h later. Embryos were collected at E17.5 and were analyzed by immunofluorescence using anti-IdU (Santa Cruz; 1:500) and anti-BrdU (Abcam; 1:10) antibodies.

Cell counts were performed after imaging, using the NIH imageJ software. Briefly, the olfactory area was first defined by its horseshoe shape in sections. Total cell numbers were counted in explants. All subsequent counts were made of positive cells within this area and expressed as a function of the total DAPI positive nuclei. Student’s *t*-test was used to calculate *p* values.

## Abbreviations

INP: Immediate neuronal precursor; E: Embryonic day; HH: Hamburger-Hamilton stage; OE: Olfactory epithelium; OP: Olfactory placode; ORN: Olfactory receptor neuron; RA: Retinoic acid.

## Competing interests

The authors declare that they have no competing interests.

## Authors’ contributions

RL, PD, and MP conceived the experiments and wrote the paper. MP performed and analyzed the experiments. LC analyzed induction markers in *Raldh* mutant mice. YM, YM, and SSM participated to immunostaining analysis on chick samples. MRB contributed to the BrdU experiments. VF performed the RARE-*lacZ* analyses. All authors read and approved the final manuscript.

## Supplementary Material

Additional file 1: Figure S1 Scheme of the main cell types of the murine olfactory epithelium. Click here for file

Additional file 2: Figure S2 Retinoic acid activity as observed in RARE-*lacZ* transgenic embryos analyzed after X-gal reactions. X-gal/*lacZ* activity is detected in the olfactory epithelium of wild-type embryos (left-side panels) at E10.5, and more weakly at E12.5, whereas it is undetectable in the olfactory region of *Raldh3*^−/−^ embryos (right-side panels; n = 4 embryos analyzed at each stage). As an internal control, *Raldh3*^−/−^ embryos exhibit RARE-*lacZ* activity in the forebrain neuroepithelium. Click here for file

Additional file 3: Table S1 Statistical data for Figure 3K and L. Click here for file
