# Supplementary figures and images for: Retinoic acid regulates olfactory progenitor cell fate and differentiation
Source: Neural Dev. 2013 Jul 5;8:13. doi: 10.1186/1749-8104-8-13 (PMC3717070; doi:10.1186/1749-8104-8-13)

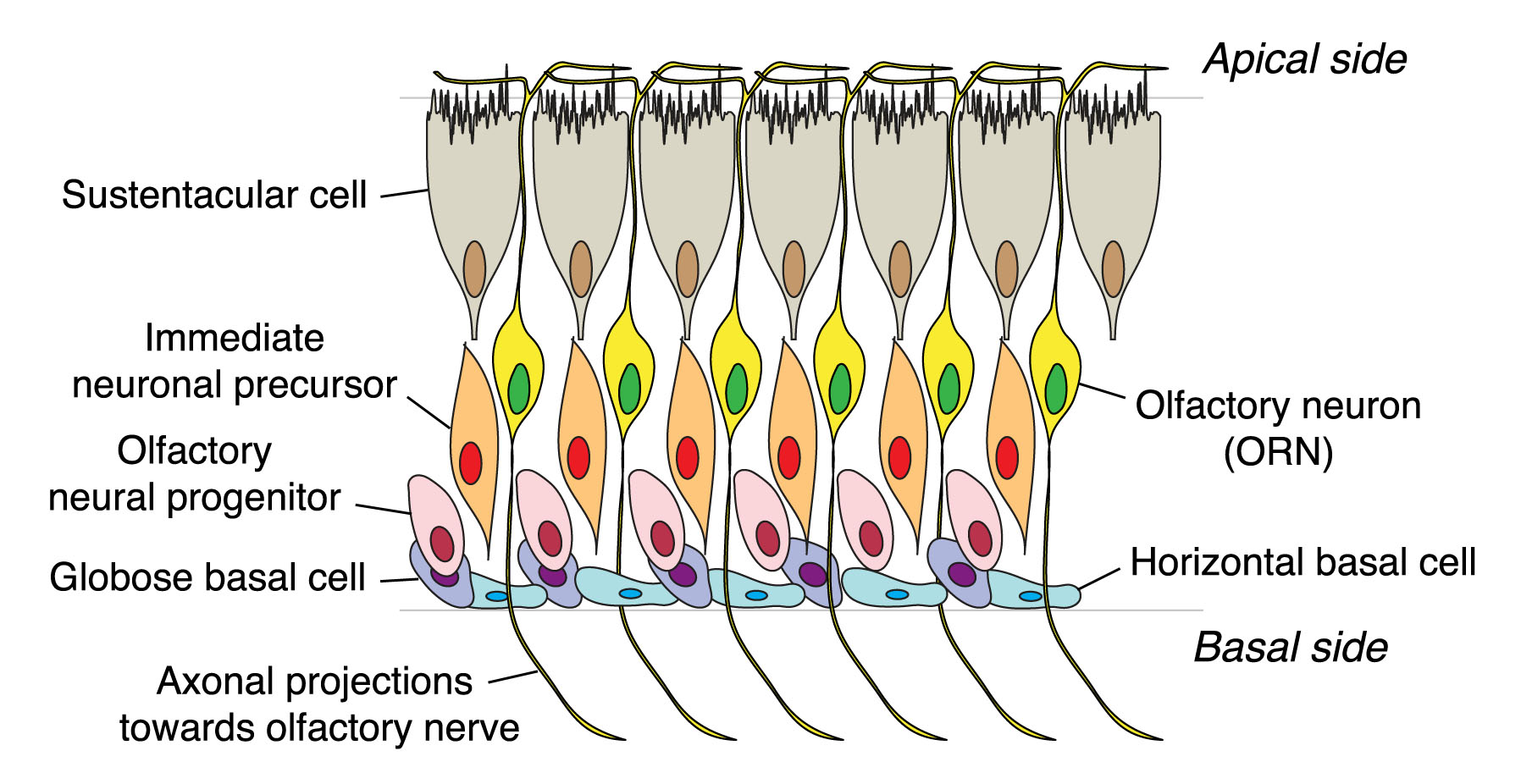

Supplement: Additional file 1: Figure S1 — Scheme of the main cell types of the murine olfactory epithelium. [file 1749-8104-8-13-S1.jpeg]

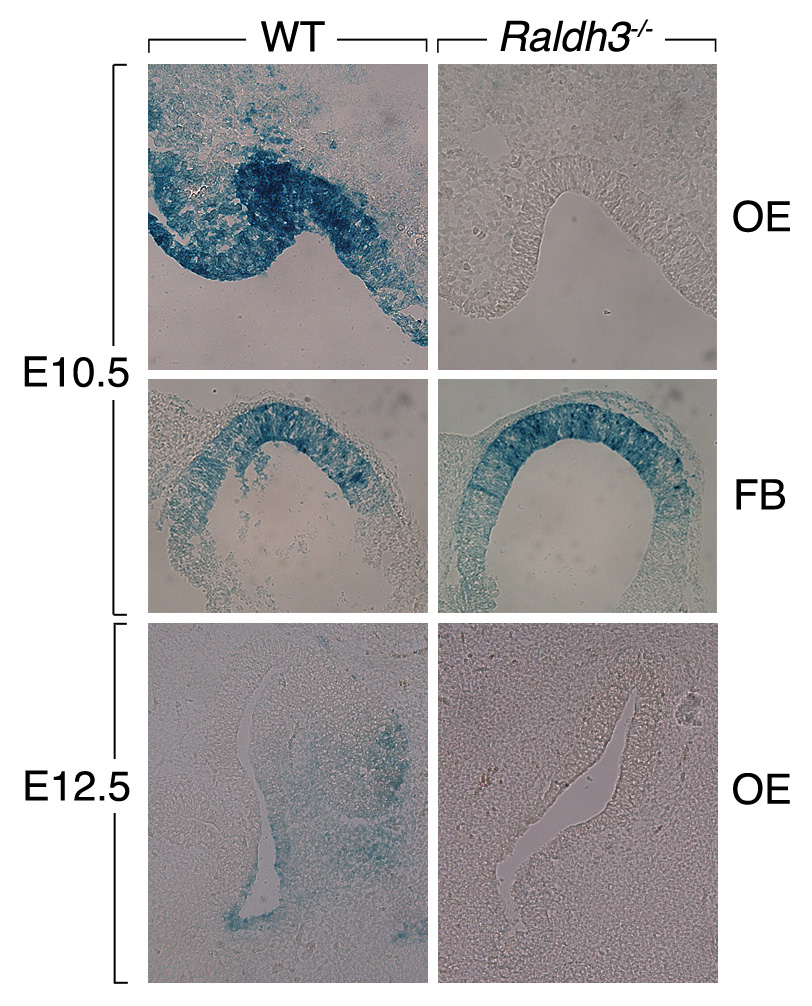

Supplement: Additional file 2: Figure S2 — Retinoic acid activity as observed in RARE-lacZ transgenic embryos analyzed after X-gal reactions. X-gal/lacZ activity is detected in the olfactory epithelium of wild-type embryos (left-side panels) at E10.5, and more weakly at E12.5, whereas it is undetectable in the olfactory region of Raldh3−/− embryos (right-side panels; n = 4 embryos analyzed at each stage). As an internal control, Raldh3−/− embryos exhibit RARE-lacZ activity in the forebrain neuroepithelium. [file 1749-8104-8-13-S2.jpeg]
