# Supplementary material for: Retinoic acid regulates olfactory progenitor cell fate and differentiation
Source: Neural Dev. 2013 Jul 5;8:13. doi: 10.1186/1749-8104-8-13 (PMC3717070; doi:10.1186/1749-8104-8-13)
Supplement: Additional file 3: Table S1 — Statistical data for Figure 3K and L. [file 1749-8104-8-13-S3.docx]

**Supplementary Table S1: statistical data for Figure 3K and L**

|  |  |  | **Ascl1** | | **Neurog1** | | **TuJ1** | |
| --- | --- | --- | --- | --- | --- | --- | --- | --- |
|  |  |  | with vit. A | without vit. A | with vit. A | without vit. A | with vit. A | without vit. A |
| **E 10.5** | *Raldh3* +/+ | AVERAGE | 5.3 ± 1.55 | 53.0 ± 4.00 | 19.0 ± 3.61 | 54.0 ± 3.61 | 0.67 ± 0.58 | 8.33 ± 1.53 |
|  |  | **p-value** | **0.00004 ***** | | **0.0002 ***** | | **0.001 **** | |
|  | *Raldh3* -/- | AVERAGE | 60.3 ± 2.52 | 63.0 ± 12.8 | 112 ± 16.6 | 88.0 ± 9.54 | 16.3 ± 1.53 | 17.0 ± 2.00 |
|  |  | **p-value** | **0.74 N.S.** | | **0.09 N.S.** | | **0.67 N.S.** | |
| **E12.5** | *Raldh3* +/+ | AVERAGE | 29.0 ± 3.61 | 3.33 ± 1.53 | 73.3 ± 4.73 | 15.7 ± 3.51 | 9.33 ± 2.52 | 48.3 ± 7.64 |
|  |  | **p-value** | **0.0003 ***** | | **0.00007 ***** | | **0.001 **** | |
|  | *Raldh3* -/- | AVERAGE | 0 | 0 | 2.67 ± 0.58 | 9.00 ± 1.00 | 58.0 ± 2.65 | 56.3 ± 6.03 |
|  |  | **p-value** | **-** | | **0.0007 ***** | | **0.68 N.S.** | |

*** p<0.001; ** p<0.01; * p<0.05

N.S. non significant

p-values calculated from 3 independent experiments by the t-Student paired test

Positive cells were normalized to DAPI positive nuclei within the same explant area.
